# Two multistate outbreaks of a reoccurring Shiga toxin-producing *Escherichia coli* strain associated with romaine lettuce: USA, 2018–2019

**DOI:** 10.1017/S0950268821002703

**Published:** 2021-12-13

**Authors:** Michelle A. Waltenburg, Colin Schwensohn, Asma Madad, Sharon L. Seelman, Vi Peralta, Sarah E. Koske, Michelle M. Boyle, Katherine Arends, Kane Patel, Mia Mattioli, Laura Gieraltowski, Karen P. Neil, Outbreak Investigation Team

**Affiliations:** 1 Centers for Disease Control and Prevention (CDC), Atlanta, Georgia, USA; 2 Epidemic Intelligence Service, CDC, Atlanta, Georgia, USA; 3 United States Food and Drug Administration, College Park, Maryland, USA; 4 California Department of Public Health, Richmond, California, USA; 5 Wisconsin Department of Health Services, Madison, Wisconsin, USA; 6 Maryland Department of Health, Baltimore, Maryland, USA; 7 Michigan Department of Health and Human Services, Lansing, Michigan, USA

**Keywords:** *Escherichia coli* (*E. coli*), food-borne infections

## Abstract

Leafy green vegetables are a common source of Shiga toxin-producing *Escherichia coli* O157:H7 (STEC O157) foodborne illness outbreaks. Ruminant animals, primarily cattle, are the major reservoir of STEC O157. Epidemiological, traceback and field investigations were conducted to identify potential outbreak sources. Product and environmental samples were tested for STEC. A reoccurring strain of STEC O157 caused two multistate outbreaks linked to romaine lettuce in 2018 and 2019, resulting in 234 illnesses in 33 states. Over 80% of patients interviewed consumed romaine lettuce before illness. The romaine lettuce was sourced from two California growing regions: Santa Maria and Salinas Valley in 2018 and Salinas Valley in 2019. The outbreak strain was isolated from environmental samples collected at sites >90 miles apart across growing regions, as well as from romaine-containing products in 2019. Although the definitive route of romaine contamination was undetermined, use of a contaminated agricultural water reservoir in 2018 and contamination from cattle grazing on adjacent land in 2019 were suspected as possible factors. Preventing lettuce contamination from growth to consumption is imperative to preventing illness. These outbreaks highlight the need to further understand mechanisms of romaine contamination, including the role of environmental or animal reservoirs for STEC O157.

## Introduction

Shiga toxin-producing *Escherichia coli* O157:H7 (STEC O157) infections cause an estimated 63 000 foodborne illnesses, 2000 hospitalisations and 20 deaths annually in the USA [1]. Most people infected with STEC O157 recover within 5–7 days; however, some infections can progress to haemolytic uremic syndrome (HUS), resulting in kidney failure and potentially death [2]. Ruminant animals, primarily cattle, are the major reservoir of STEC O157, but the pathogen has also been found in other animals, including pigs, birds and other wildlife [3–5].

Leafy green vegetables such as lettuce are the second most common source of foodborne STEC O157 illness outbreaks in the USA, especially outbreaks occurring in the fall [6, 7]. Most leafy green production in the USA occurs outdoors in California and Arizona, with California farms producing approximately 70% of US lettuce and leafy greens [8]. California contains multiple leaf lettuce growing regions, which are areas of land devoted to the growth and harvest of lettuce [9]. Two primary areas are the Salinas Valley and Santa Maria growing regions of California. The Salinas Valley growing region encompasses Monterey, Santa Clara, Santa Cruz and San Benito counties, while the Santa Maria growing region is comprised of San Luis Obispo and Santa Barbara counties [10]. These regions have also been referred to by the US Food and Drug Administration (FDA) as the California Central Coast growing region [11].

Whole genome sequencing (WGS) is facilitating the identification of reoccurring, emerging and persisting strains of enteric bacteria. Reoccurring strains are sets of genetically related bacteria that cause periodic outbreaks of illnesses separated by periods where the strain is not identified or occurs at very low levels. Before 2018, a rare strain of STEC O157 was identified by WGS in isolates from patients in a suspected leafy green-associated outbreak that occurred in the fall of 2017 [12]. During 2018–2019, local, state and federal officials investigated two large multistate illness outbreaks caused by this reoccurring strain of STEC O157 that were linked to romaine lettuce from two large growing regions in California. We describe the epidemiology, laboratory and traceback investigations that linked these two outbreaks to the consumption of romaine lettuce, and the public health implications of the investigation findings.

## Methods

### Outbreak detection and case identification

State and local public health laboratories routinely perform molecular-based subtyping of clinical isolates that are received from clinical laboratories and submit the resulting molecular fingerprints to PulseNet, the national molecular subtyping network for foodborne disease surveillance [13]. PulseNet surveils for temporal clusters of infections with the same strain of bacteria, which might represent outbreaks linked to a common exposure. The Centers for Disease Control and Prevention (CDC) and state and local health departments initiate multistate investigations to determine the cause of the outbreak and prevent additional illnesses.

For both investigations, PulseNet was used to detect the outbreaks and identify additional cases. Before 2019, PulseNet used pulsed-field gel electrophoresis (PFGE) as the main subtyping method to detect clusters of STEC [14]. In 2019, PulseNet laboratories began using WGS analysis as the primary method for the detection of STEC outbreaks, performing core genome multi-locus sequencing typing (cgMLST) to determine relatedness between STEC O157 isolates using standard methods. WGS was used as an additional subtyping method in outbreaks for several years before that [15, 16]. PulseNet considered a threshold of 10 allele differences for initial cluster detection of related STEC isolates by cgMLST [17]. Epidemiologists used this information to establish a case definition for each investigation. Once a cluster was identified, the appropriate allele range for the case definition was continually assessed as potential new isolates were identified, considering factors such as the clonality of the strain and the tightness of the original allele range [15, 17]. As a result, allele ranges may have increased as new isolates were identified.

### Hypothesis generation and epidemiological investigation

Multiple hypothesis-generating strategies were used during these investigations, including routine state enteric disease questionnaires and the CDC national hypothesis-generating questionnaire, which is a set of questions on various food, animal and other exposures in the week prior to illness onset. Based on initial exposure information showing multiple patients reportedly consuming leafy greens, state and local officials asked patients additional detailed questions about leafy greens, including purchase locations and restaurants visited in the week before illness onset. Food exposures among patients were compared with expected rates for foods reported by healthy people in the 2006–2007 Foodborne Diseases Active Surveillance Network (FoodNet) population survey [18]. This survey contains data on foods consumed by healthy people in the seven days before interview and aids in hypothesis generation by identifying food exposures among patients that are higher than expected using a binomial probability distribution.

### Product testing, traceback investigation and field investigations

State and local investigators collected available leftover products of interest from patients' homes. Products were tested for STEC at state laboratories and further characterised by WGS if any STEC isolates were identified.

State and local health departments, state regulatory agencies and FDA conducted traceback investigations to determine whether the products of interest came from a common source or a point of contamination in the supply chain [19]. Traceback activities focused on individuals who provided detailed information about their romaine purchase histories and/or illness subclusters, defined as locations (e.g., restaurants, grocery stores) where two or more unrelated patients reported eating or purchasing suspected food items in the week before becoming ill. Geographic diversity was also considered when selecting individuals to include in the traceback investigation. State partners collected information on locations, menu items, meal/purchase dates, invoices and product distribution records regarding restaurant subclusters. Individual purchase histories, loyalty card records and receipts were collected for grocery store subclusters, with permission from patients. FDA used this information to determine whether leafy greens consumed by patients came from a common entity (e.g., distributor, processor and/or farm).

On-site farm investigations based on traceback findings were conducted to evaluate source(s) and routes of STEC contamination from the environment to the lettuce [19–21]. The investigations included on-farm and adjacent land observations and environmental sampling. Water, sediment, soil, cattle faeces, scat and other environmental samples collected during on-site farm investigations were cultured for STEC non-O157 and/or STEC O157 specifically; resulting isolates were genetically characterised by WGS [22].

## Results

### STEC O157 outbreak: USA, October–December 2018

#### Outbreak detection and case identification

**Fig. 1.**
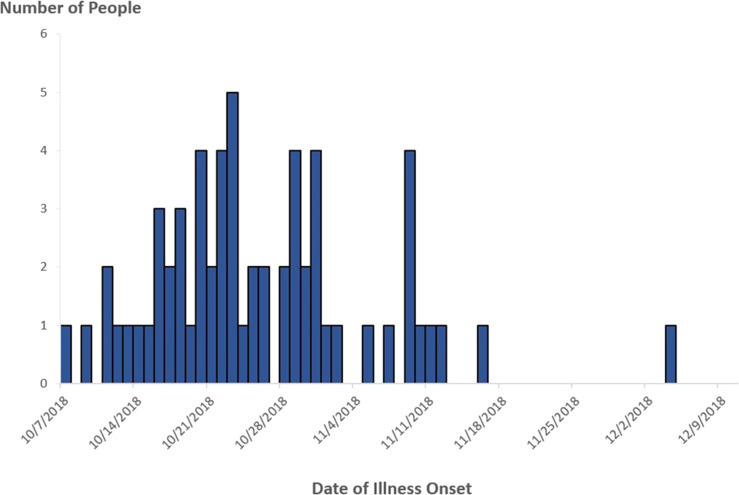
People infected with the outbreak strain of STEC O157 (*n* = 62), by date of illness onset*, USA, October–December 2018. *Some illness onset dates have been estimated from other reported information.

In November 2018, PulseNet detected a cluster of 10 STEC O157 infections with two very similar PFGE patterns. Additional infections with similar PFGE patterns that were also confirmed to be related by WGS were added to the cluster as the investigation progressed. A case was defined as infection with STEC O157 with any of the 10 PFGE pattern combinations comprising the outbreak strain, with illness onset occurring between 7 October 2018 and 4 December 2018 (Fig. 1).

**Fig. 2.**
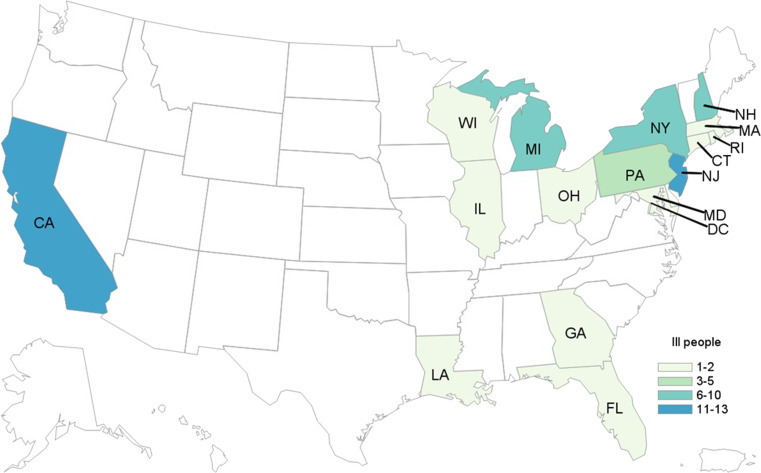
People infected with the outbreak strain of STEC O157 (*n* = 62), by state of residence, USA, October–December 2018.

In total, 62 cases were identified from 16 states and the District of Columbia (Fig. 2). Patients ranged in age from <1 to 84 years (median: 25), and 66% were female. Among 54 patients with available information, 25 (46%) were hospitalised. Among 50 patients with information, two adults developed HUS. No deaths were reported. All outbreak isolates were closely related genetically by WGS (0–4 alleles different).

#### Hypothesis generation and epidemiological investigation

Among 36 patients with available exposure information, 30 (83%) reported consuming romaine lettuce in the week before illness onset, which was significantly higher when compared to 47% of healthy people in the FoodNet Population Survey (*P* < 0.01). Investigators in California and Michigan identified two restaurant subclusters, which included two and four patients, respectively. In total, four (67%) patients who ate at these restaurants reported consuming salads containing romaine lettuce before illness onset.

#### Product testing, traceback investigation and field investigations

Traceback was initiated from six points of service associated with eight patients from four states, including the illness subclusters in California and Michigan. Traceback identified 13 distributors, 17 farms and 15 ranches in three counties in the Salinas Valley and Santa Maria growing regions of California as potentially supplying romaine lettuce that was consumed during this outbreak [19]. Product traceback efforts by FDA and state and local officials did not identify a single grower or processor of romaine that could explain the entire outbreak.

During November–December 2018, FDA, CDC and/or state partners conducted on-site investigations at three farms identified by the traceback investigation. During an investigation on a farm in Santa Barbara County that was identified in multiple legs of the traceback investigation, investigators noted that the land adjacent to the farm was used for animal grazing by cattle and horses and observed extensive wild animal activity near an agricultural water reservoir used for irrigation and harvest/post-harvest of romaine lettuce on the farm [20]. The outbreak strain of STEC O157 was isolated from a sediment sample taken from the untreated agricultural water reservoir. The sediment isolate and clinical isolates were closely related genetically (0–4 alleles different). Investigators determined the most likely way romaine on this farm became contaminated was from the use of the agricultural water reservoir, either through direct application of the water to romaine or through cross-contamination from the use of the water on harvest equipment [20]. However, investigators were not able to identify a definitive faecal source of STEC to the reservoir or the route(s) of contamination of the agricultural water reservoir to the lettuce. The outbreak strain was not detected in more than 150 other samples collected during on-site investigations at the three farms, including soil, animal excreta and other agricultural water samples [20].

#### Public health impact

By 20 November 2018, the epidemiological evidence had identified romaine lettuce as the food vehicle responsible for the outbreak; however, the traceback investigation had not yet identified the exact growing locations of romaine lettuce consumed by patients. Based on strong epidemiological evidence, the rapidly increasing number of illnesses, and the concern that contaminated romaine lettuce remained on the market and in homes, CDC and FDA advised consumers and retailers not to eat, sell or serve any romaine lettuce [23, 24]. By 26 November 2018, based on additional traceback information received and analysed, the public health advisory previously issued by CDC and FDA was narrowed in scope to pertain to only romaine from specific California growing regions. On 13 December 2018, the scope of the public health advisory was further narrowed to specific California counties based on subsequent traceback investigation findings. Additionally, the Santa Barbara County farm with the sediment sample from the water reservoir that yielded the outbreak strain recalled all products that may have come into contact with water from the reservoir during the time period of interest due to potential contamination, including red leaf lettuce, green leaf lettuce and cauliflower [25].

### STEC O157 outbreak: USA, September–December 2019

#### Outbreak detection and case identification

**Fig. 3.**
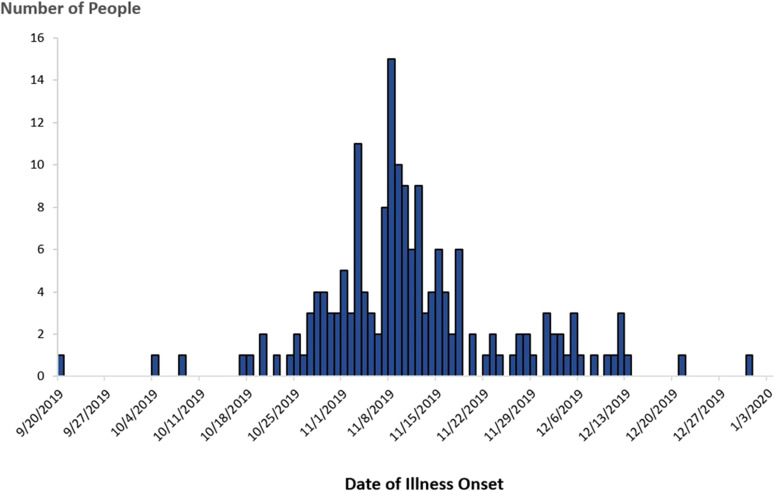
People infected with the outbreak strain of STEC O157 (*n* = 172), by date of illness onset*, USA, September–December 2019. *Some illness onset dates have been estimated from other reported information.

In November 2019, PulseNet detected a cluster of five STEC O157 infections caused by bacteria that were closely related to one another by WGS (within 0–1 allele differences) and a multistate investigation was initiated. Isolates in this cluster were also related genetically to isolates in the 2018 investigation within 0–6 allele differences. A case was defined as an infection with STEC O157 closely related to the outbreak strain by WGS, with illness onset occurring between 20 September 2019 and 31 December 2019 (Fig. 3).

**Fig. 4.**
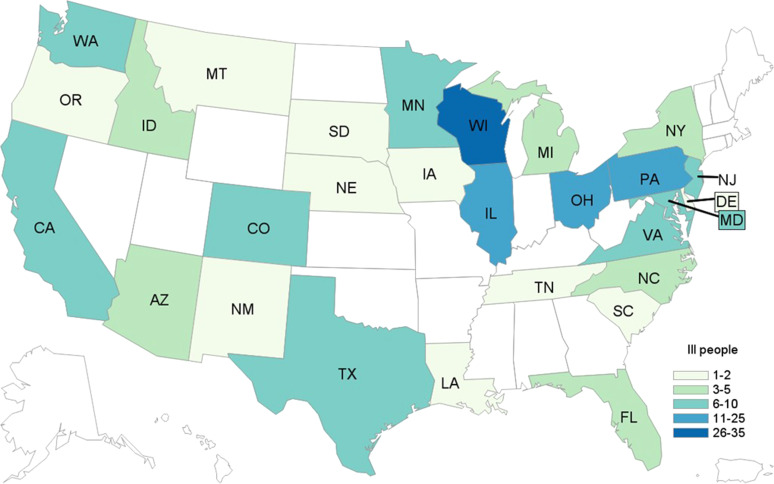
People infected with the outbreak strain of STEC O157 (*n* = 172), by state of residence, USA, September–December 2019.

In total, 172 cases were identified from 28 states (Fig. 4). Patients ranged in age from <1 to 89 years (median: 27), and 65% were female. Among 169 patients with available information, 88 (52%) were hospitalised. Among 160 patients with information, 16 developed HUS, and eight of these were children <18 years of age. No deaths were reported.

All outbreak isolates were related genetically by WGS (0–7 alleles different per cgMLST analysis). When compared with outbreak isolates from the 2018 investigation, all isolates from both outbreaks were related genetically by WGS within 0–8 allele differences. Within this reoccurring strain, the isolates within each outbreak clustered more closely with one another; isolates from the 2018 outbreak were within 0–4 allele differences of each other (median: 1 allele difference) while isolates from the 2019 outbreak were within 0–7 allele differences of each other (median: 1 allele difference).

#### Hypothesis generation and epidemiological investigation

The investigation was rapidly narrowed to leafy green vegetables within six days of initiating the investigation based on epidemiological evidence coupled with the history of this reoccurring strain linked to prior leafy green outbreaks. Among 114 patients with information available, 95 (83%) reported consuming romaine lettuce in the week before illness onset, which was significantly higher when compared to 47% of healthy people in the FoodNet Population Survey (*P* < 0.01). Investigators in Maryland identified five patients who all consumed the same brand and type of a single-serve prepackaged salad bowl purchased from the same retail chain. Romaine lettuce was the only type of lettuce within the salad, and the source of romaine was labelled as the Salinas Valley growing region of California.

#### Product testing, traceback investigation and field investigations

Investigators collected 11 leftover romaine lettuce-containing products from patient homes in five states, and testing isolated the outbreak strain of STEC O157 from two products. In November 2019, one of the patients in Maryland who reported consuming the common brand of single-serve prepackaged salad bowl had an unopened leftover salad bowl. The Maryland Department of Health tested this unopened package and isolated the outbreak strain from the romaine lettuce. In December 2019, the Wisconsin Department of Health Services, the Wisconsin Department of Agriculture, Trade and Consumer Protection, Bureau of Laboratory Services and the Wisconsin State Laboratory of Hygiene collected and tested a different brand of an unopened romaine-containing salad collected from a patient's home and isolated the outbreak strain from the romaine lettuce. Both products were labelled as containing romaine from the Salinas Valley growing region of California.

Traceback was initiated from 15 points of service associated with 15 patients from seven states, which included the stores where the two products that yielded the outbreak strain in both Maryland and Wisconsin were purchased. Traceback identified 13 growers and multiple fields in various counties in the Salinas Valley growing region of California that potentially supplied romaine lettuce that was contaminated and consumed during this outbreak. Traceback also identified three growers in Mexico that supplied one of the distributors, but the Mexican growers were not sole suppliers of romaine to any of the points of service included in the traceback [19]. Product traceback efforts by FDA and state and local officials did not identify a single grower or processor of romaine that could explain the entire outbreak.

After the traceback investigation of patient exposures and product samples from Maryland and Wisconsin identified romaine lettuce from the Salinas Valley growing region of California as the likely outbreak vehicle, FDA, CDC and/or California state partners conducted on-site investigations at three farms in this growing region during November 2019–February 2020. Investigators noted cattle grazing and cattle feeding operations on lands adjacent to several growing fields identified during the traceback investigation. Investigators also observed evidence of wild animal activity near romaine fields. Testing isolated the outbreak strain of STEC O157 from a cattle faecal-soil composite sample collected from public land in Monterey County. The composite sample was within two miles upslope from a produce farm identified during the traceback investigation [21]. Laboratory testing also identified a non-outbreak strain of STEC O157 from a water sample from the Salinas River and identified multiple strains of non-O157 STEC from 12 other water, soil, cattle faeces or compost sample tests. Investigators determined the most likely way romaine on these farms became contaminated was from adjacent land use for cattle grazing [21]. However, the investigation could not determine a definitive faecal source or the route(s) of contamination of the romaine fields.

Shortly after this outbreak was identified, FDA, CDC and California state partners revisited the farm in Santa Barbara County, where in 2018, the reoccurring outbreak strain was isolated from a reservoir sediment sample. The visit was part of a planned follow-up from 2018 activities, which was expedited due to the history of this recurrent strain. Well water, reservoir water and reservoir sediment were collected from the farm and tested for the presence of STEC O157 [22]. STEC O157 was not detected in any of the on-farm water or sediment samples. Recently deposited cattle faeces and water from a cattle trough were collected from a cow-calf operation on land adjacent to this farm. STEC O157 was isolated from the cattle faeces and water trough, and all isolates were closely related genetically to the sediment sample from the agricultural water reservoir associated with the 2018 outbreak by WGS (within 0–2 allele differences). The farm in Santa Barbara County where the outbreak strain was isolated in 2018 was not identified during the 2019 traceback investigation.

#### Public health impact

On 21 November 2019, the distributor of the product that yielded the outbreak strain in Maryland recalled a variety of prepackaged salad bowls [26]. By 22 November 2019, epidemiological, laboratory and traceback evidence confirmed romaine lettuce from the Salinas Valley growing region in California as the food vehicle for the outbreak. CDC and FDA advised consumers and retailers not to eat, sell or serve romaine lettuce harvested from the Salinas Valley California growing region [27, 28].

## Discussion

Over the past decade, 31 STEC outbreaks linked to leafy greens have occurred in the USA; the two recent outbreaks of STEC O157 discussed here demonstrate the continued risk of romaine lettuce as a cause of illness [7]. Despite the romaine lettuce implicated in these two outbreaks being harvested from two different growing regions in California, WGS was used to confirm that isolates from both outbreaks were closely related genetically. Since romaine lettuce is typically consumed raw, preventing contamination from growth to consumption is imperative to preventing foodborne illness.

Clear, actionable and timely risk communication is essential during foodborne illness outbreaks to prevent additional illnesses. Leafy green outbreaks have unique features that make clear and actionable risk communication difficult. The complicated nature of epidemiological and traceback investigations during leafy green outbreaks can delay or prevent the identification of a specific product linked to illness [19]. For instance, due to the concern regarding the rapidly increasing number of illnesses and severity of illnesses in the 2018 outbreak investigation, public health and regulatory agencies initially issued broad national advisories against eating any romaine lettuce before a more specific source of the contaminated romaine lettuce had been identified. Although advice to consumers and retailers was eventually narrowed to avoid romaine lettuce from specific California counties, potentially contaminated lettuce was challenging for consumers and retailers to identify, as most products were not labelled with growing region information.

In response to challenges encountered during the 2018 investigation, FDA requested industry to voluntarily label harvested romaine lettuce with date and growing region to more quickly identify potentially contaminated products in the case of future outbreaks, and so that consumers and retailers can more readily identify where lettuce was grown [10]. The voluntary labelling changes made by industry facilitated traceback efforts during the 2019 outbreak investigation by providing preliminary information as to which growing region may have produced the contaminated romaine lettuce, which was verified by traceback documentation. Specific and actionable advice was quickly given to consumers and retailers due to the growing region labels, which may have prevented additional illnesses. However, challenges were still evident given that multiple growing locations were included on some product labels. There was also potential for confusion regarding what locations indicated on packaging were considered part of the Salinas Valley growing region.

These investigations highlight the usefulness of WGS in foodborne outbreak investigations. First, WGS can be used to establish links between ongoing illness outbreaks and historic outbreaks caused by closely related strains, which can help guide hypothesis generation by identifying potential foods of interest. In the 2019 investigation, knowing that the outbreak strain was a reoccurring strain that caused recent leafy green-associated outbreaks, coupled with patients commonly reporting leafy greens in initial interviews, enabled us to rapidly narrow the focus of the investigation to romaine and other leafy greens, more quickly assess leafy green exposures of interest, and gather detailed information about these products.

The use of WGS analysis also provided support for romaine lettuce as the source of each outbreak by helping to link the bacteria identified in patients to the bacteria identified in environmental or product testing. In the 2018 outbreak investigation, STEC O157 found in an environmental sample from a romaine grower identified by the traceback investigation was closely related to STEC identified in patients included in the outbreak, which helped inform the scope of products recalled. In the 2019 investigation, WGS analysis of STEC O157 in romaine lettuce-containing salads linked to the Maryland and Wisconsin patients helped to confirm romaine lettuce as the source of the outbreak. These outbreaks also serve to highlight that while WGS can be a powerful tool to aid in foodborne outbreak investigations, WGS results must always be interpreted in conjunction with epidemiological and traceback evidence when evaluating whether a certain food or producer is the source of an outbreak. During the 2019 investigation, the reoccurring strain was identified in the environment (i.e., cattle faeces, water trough) during follow-up testing near the same grower in Santa Barbara County that was linked to the 2018 outbreak. However, epidemiological and traceback evidence did not identify this grower as having supplied romaine linked to patients included in the 2019 outbreak.

These outbreaks highlight the need to further understand mechanisms of lettuce contamination during growth, processing and harvesting of leafy greens generally and romaine specifically. Two common factors identified during field investigations in both outbreaks included land use for cattle grazing and wild animal activity, such as flocking birds, adjacent to romaine fields and/or agricultural water reservoirs. However, investigators could not definitively determine how romaine lettuce became contaminated. Given that cattle are the major reservoir of STEC O157, a plausible source of STEC O157 contamination could be from cattle through adjacent land use. STEC O157 shed in animal faeces can contaminate the environment directly or indirectly through water runoff, wind or other animals [29–31]. Wild animals that carry STEC O157, such as rodents and flocks of birds, such as Starlings, were observed near romaine fields and could have also been sources of STEC O157 and/or vectors from the cattle directly to the produce or indirectly to water sources [3, 5, 32]. Additionally, seasonality and practices associated with other adjacent land entities or shared vehicle and equipment roadways are important to understand when considering routes of contamination, acknowledging potential interactions between factors.

One outstanding question is how this reoccurring strain of STEC O157 could contaminate romaine grown across two large growing regions in California. The locations where the outbreak strain was found in environmental samples during the 2018 and 2019 investigations are over 90 miles apart. One possible explanation is that zoonotic or environmental reservoirs spread this genomic strain to both growing regions. Cattle that harbour this genomic strain of STEC O157 might be moved between regions and subsequently introduce the strain to different areas. Other animals that migrate between regions and are known STEC O157 hosts, such as large flocks of wild birds, might also contribute to the spread of this strain [5, 32]. Equipment shared between regions could also potentially explain the presence in different areas, although this is less likely.

However, while any of the above factors could contribute to the introduction of this strain to both growing areas, the WGS analysis indicates that more complex factors could be at work. cgMLST analyses suggest that while all the isolates in this reoccurring strain are closely related genetically, the isolates from the 2018 and 2019 outbreaks fall into two genetic groupings that are independently more closely related to themselves than to one another. While additional WGS analyses are needed to evaluate whether the genetic differences between these two potential groupings are meaningful, it does appear to correlate with investigational data: the genetic group from the 2018 outbreak traced back to the Santa Maria and Salinas Valley growing regions while the genetic group from the 2019 outbreak traced back to the Salinas Valley growing region [21]. These potential genetic groupings within the overall reoccurring strain, which align with growing region, suggest that a single, common source reservoir ‘seeding’ both the Santa Maria and Salinas Valley growing regions with the same strain might be an overly simplistic explanation for the mechanism of the persistence and spread of STEC O157 in animals and/or the environment. Alternatively, the genetic groupings could represent adaptations of the strain in different host reservoirs (e.g., cattle *vs.* wild birds *vs.* other animals) or other genetic adaptations influenced by local ecologic factors after an initial introduction of the strain to each region. Conducting additional research to better understand the ecology and movement of STEC across agricultural regions may be beneficial.

These large, reoccurring multistate outbreaks caused by romaine lettuce indicate a continued risk to consumers and the need for further efforts to understand mechanisms of on-farm contamination to prevent product contamination from growth to consumption. In response to these and other outbreaks, FDA released its 2020 Leafy Greens STEC Action Plan, which identified three key priority areas, namely prevention, response and addressing knowledge gaps [33]. FDA and state partners have prioritised routine inspections and on-farm readiness reviews for leafy greens farms covered by the Produce Safety Rule [34]. Additionally, a longitudinal study of the ecology of human pathogens in the environment has been initiated in California, focusing on how human pathogens survive, move and possibly contaminate produce prior to harvest. Efforts to enhance traceability systems and refine industry product labelling could further assist with outbreak-related communications and traceback efforts. The potential role of environmental or animal reservoirs for STEC O157 requires broader industry-wide research and control measures to prevent contamination, which is imperative to preventing foodborne illness linked to romaine lettuce, as it is typically consumed raw.

## Data Availability

The data that support the findings of this study are available from the corresponding author on reasonable request.
